# Body Fineness Ratio as a Predictor of Maximum Prolonged-Swimming Speed in Coral Reef Fishes

**DOI:** 10.1371/journal.pone.0075422

**Published:** 2013-10-18

**Authors:** Jeffrey A. Walker, Michael E. Alfaro, Mae M. Noble, Christopher J. Fulton

**Affiliations:** 1 Department of Biological Sciences, University of Southern Maine, Portland, Maine, United States of America; 2 Department of Ecology and Evolutionary Biology, University of California, Los Angeles, California, United States of America; 3 ARC Centre of Excellence for Coral Reef Studies, Research School of Biology, The Australian National University, Canberra, Australian Capital Territory, Australia; University of Hull, United Kingdom

## Abstract

The ability to sustain high swimming speeds is believed to be an important factor affecting resource acquisition in fishes. While we have gained insights into how fin morphology and motion influences swimming performance in coral reef fishes, the role of other traits, such as body shape, remains poorly understood. We explore the ability of two mechanistic models of the causal relationship between body fineness ratio and endurance swimming-performance to predict maximum prolonged-swimming speed (*U_max_*) among 84 fish species from the Great Barrier Reef, Australia. A drag model, based on semi-empirical data on the drag of rigid, submerged bodies of revolution, was applied to species that employ pectoral-fin propulsion with a rigid body at *U*_max_. An alternative model, based on the results of computer simulations of optimal shape in self-propelled undulating bodies, was applied to the species that swim by body-caudal-fin propulsion at *U_max_*. For pectoral-fin swimmers, *U_max_* increased with fineness, and the rate of increase decreased with fineness, as predicted by the drag model. While the mechanistic and statistical models of the relationship between fineness and *U_max_* were very similar, the mechanistic (and statistical) model explained only a small fraction of the variance in *U_max_*. For body-caudal-fin swimmers, we found a non-linear relationship between fineness and *U_max_*, which was largely negative over most of the range of fineness. This pattern fails to support either predictions from the computational models or standard functional interpretations of body shape variation in fishes. Our results suggest that the widespread hypothesis that a more optimal fineness increases endurance-swimming performance via reduced drag should be limited to fishes that swim with rigid bodies.

## Introduction

**Figure 1:**
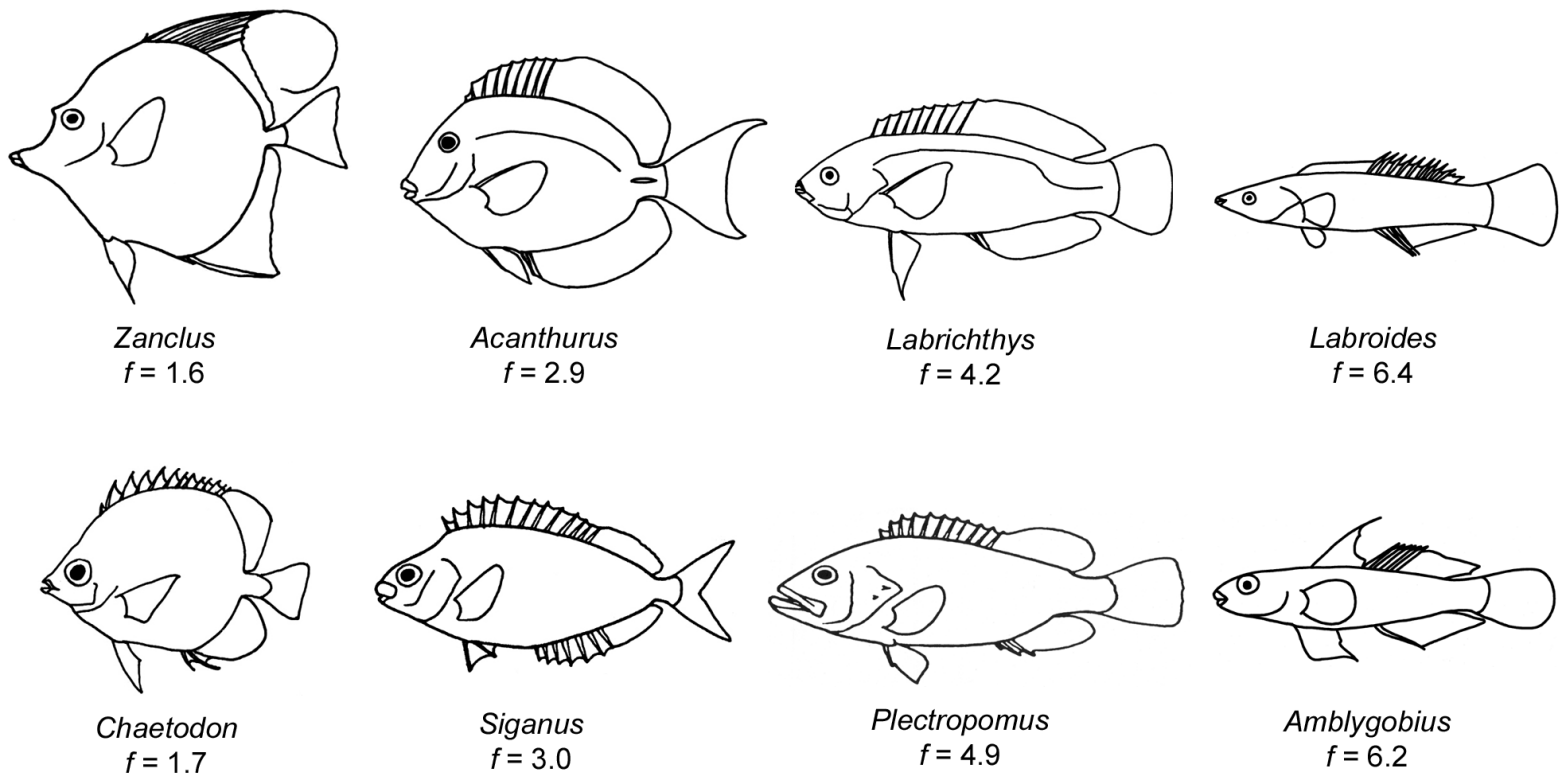
Body shape variation of fishes sampled in this study. The examples show the range of fineness in both pectoral fin (MPF, top row) and body and caudal fin (BCF, bottom row) swimmers.

The ability to achieve high prolonged-swimming speeds is an important factor limiting access to resources in the high-energy zones of reefs and, as a consequence, variation in this trait can strongly influence the structure of reef fish communities [1]–[3] and the evolution of the underlying morpho-physiological (M-P) traits that determine endurance-swimming performance [4]. Numerous, candidate M-P traits potentially affect endurance-swimming performance. While we have gained insights into how fin shape can influence swimming speed performance in coral reef fishes [2], [5], [6], we lack similar understanding of the possible consequences of their body shape diversity [4], [7], [8]. The bodies of the conspicuous fishes swimming on a coral reef range from laterally flattened discs to elongated, fusiform hulls (Fig. 1), an axis of variation that is effectively captured by the fineness ratio (a measure of how elongate a fish is relative to its transverse sectional diameter). The perceived association between body fineness and position above the reef suggests a causal effect of fineness on endurance-swimming performance and, ultimately, the ability to inhabit the reef's high-energy zones [9]. Here, we combine causal modeling with the comparative method to test the putative causal effect of fineness on a measure of endurance-swimming performance, the maximum prolonged-swimming speed [10], [11] in a community of coral reef fishes from the Great Barrier Reef, Australia.

The propulsive mechanism of the conspicuous fishes on a coral reef can be divided into those that power the entire range of prolonged-swimming speeds using oscillating median and/or pectoral fins (MPF swimmers) and those that power the higher end of the prolonged-swimming speed range with body and caudal fin undulation (BCF swimmers). For the MPF swimmers we use a model in which maximum prolonged-swimming speed increases as a function of decreased drag on the body. This “drag model” assumes that the source of drag (the body) and the source of thrust (the pectoral fins) are distinct, so that the only force on the body relevant to optimizing fineness is in the direction opposite the swimming direction. The drag that a biological or human-engineered motor has to overcome to swim or fly is termed parasite drag. Some version of the drag model is frequently used to understand body shape variation in fishes [12]–[22]. Within this literature, it is commonly stated that the optimal fineness for endurance swimming is 4.5, a value which we refer to as the traditional drag model. Our employment of the drag model differs from these previous uses by 1) refining the function of drag on fineness, 2) explicitly modeling the relationship between drag and swimming performance, and 3) limiting the model to MPF swimmers.

In fishes that swim using body-and-caudal-fin undulation (the BCF swimmers), the sources of drag and thrust are not distinct since the body is self-propelled (i.e. not propelled by an attached fin). Consequently, the drag model would not seem to be a particularly fruitful model to understand body shape variation in BCF swimmers despite its frequent use for this purpose [12]–[22]. For fishes the BCF swimmers, we do not have a model that makes precise quantitative predictions. Instead, we generate a qualitative relationship between fineness and endurance-swimming performance using two published, computational models of the effect of body shape on endurance swimming performance in self-propelled undulating bodies [23], [24]. Importantly, the computational models account for forces with components directed behind the fish (drag), in the direction of swimming (thrust), and in directions normal to thrust-drag axis. While only drag removes kinetic energy from the moving fish, the normal forces contribute (with drag) to the wasted energy total and thus reduce mechanical efficiency. Both thrust and normal components are important to modeling how fineness affects prolonged-swimming performance since the latter is a function of speed and efficiency. We refer to the predictions generated from these computational models as the “drag-thrust model”.

The drag and drag-thrust models essentially make the same general prediction: maximum prolonged-swimming speed will increases with fineness, at least through the range of fineness in our sample. Our drag model further predicts how the effect of fineness on performance will weaken as fineness increases. Our drag-thrust model is not more precise on the shape of the relationship between fineness and maximum prolonged-swimming speed because the computational work to derive this shape has not been done. We test our predictions using species-means of both performance and morphometric traits collected from a community of coral reef fishes from the Great Barrier Reef, Australia, an assemblage that has developed into an important system for integrating laboratory swimming performance measures into patterns of ecology [1]–[3], [25]–[28]. Because our dataset is comprised of a diverse range of species all measured in the same laboratory with the same methodology, our broad-scale comparison does not suffer from inter-lab variance inherit in studies that have compiled data from the literature [29]. Additionally, our comparison of swimming performance in real fishes provides a biological complement [30] to the computational modeling studies of fish body shape-swimming performance relationships that have recently become available [23], [24], [31], [32]. The drag model explains a very small amount of variation in endurance-swimming performance and we discuss the implications of this. Our BCF results are inconsistent with the drag-thrust model but consistent with other published results at more fine-grained taxonomic scales.

## Materials and Methods

### Ethics statement

This study was carried out in strict accordance with the protocols approved by the James Cook University Animal Experimentation Ethics Committee (A656-01). All efforts were made to minimize animal suffering through careful collection, handling, and swimming trials based upon the natural rheotaxic behaviour and self-motivation of individuals.

### Derivation of the drag model for pectoral fin swimmers

The drag model has two components: the relationship between body shape and drag and the relationship between drag and endurance-swimming performance. We model the relationship between drag and endurance-swimming performance using Froude efficiency, which is 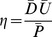 for a motor propelling a rigid body, where 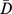, 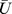, and 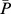 are the total drag on the body, speed of the body, and total (motor) power averaged over the stroke cycle. The drag model is a rearrangement of Froude efficiency:
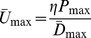
(1)where, for a fish, *max* refers to maximum aerobic power and the maximum speed and drag attainable given this power. The parasite drag on a body moving at maximum speed is 
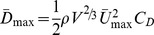
, where *ρ* is the density of the fluid, *V* is the volume of the body, and *C*_D_ is the unitless, volume-specific drag coefficient. Substituting into eq. 1 and re-arranging, we have
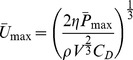
(2)The function of *C*_D_ on fineness, *C*_D_(*f*), has been used to make general predictions on how swimming performance should vary with fineness without explicitly relating endurance swimming performance to *C*_D_ [12]–[22]. We explicitly parameterize eq. 2 to generate very specific predictions of the effect of fineness on maximum prolonged swimming speed for the MPF swimmers moving in the laminar (Reynolds number from from 10^4^ to 10^5^) and transitional (Reynolds number from 10^5^ to 10^6^) flow regimes.

We model *C*_D_(*f*) using equations of semi-empirical estimates of drag upon rigid bodies of revolution (any body with rotational symmetry about the long axis) submerged in a fluid flowing at constant velocity [33]. For an elongate body of revolution, fineness, *f*, is the ratio of length to maximum cross sectional diameter. For a fish with an elliptical cross-section, *f* is often defined as ratio of standard length to body depth, a measure that does not account for varying flattening or eccentricity of the ellipse. We follow Lighthill [34] and Walker [35] and define fineness in fish as the ratio of standard length to the equivalent diameter of a circle with the same perimeter or area as the maximum cross-section of the fish (details are given below).

The total drag on a submerged body of revolution in a uniform flow is the sum of skin friction and pressure drag components. Skin friction drag is a force tangential to the body surface that occurs because of inter-molecular interactions arising from water molecules sliding past each other and the body surface. This friction creates a velocity gradient (normal to the surface) in a thin region around the body (the boundary layer). At the Reynold's number, *Re*, relevant to the fish in this study, the boundary layer will likely contain laminar flow [36]. Pressure (or form) drag arises because of spatial variation in the distribution of pressure normal to the surface. For a rigid body in a laminar flow, the largest source of pressure drag is due to the separation of the boundary layer, which creates a region of low pressure downstream of the point of separation. To compare the drag on a shape independent of scale, speed, and fluid density, drag and drag components are typically normalized as a drag coefficient *C*_D_.

**Figure 2:**
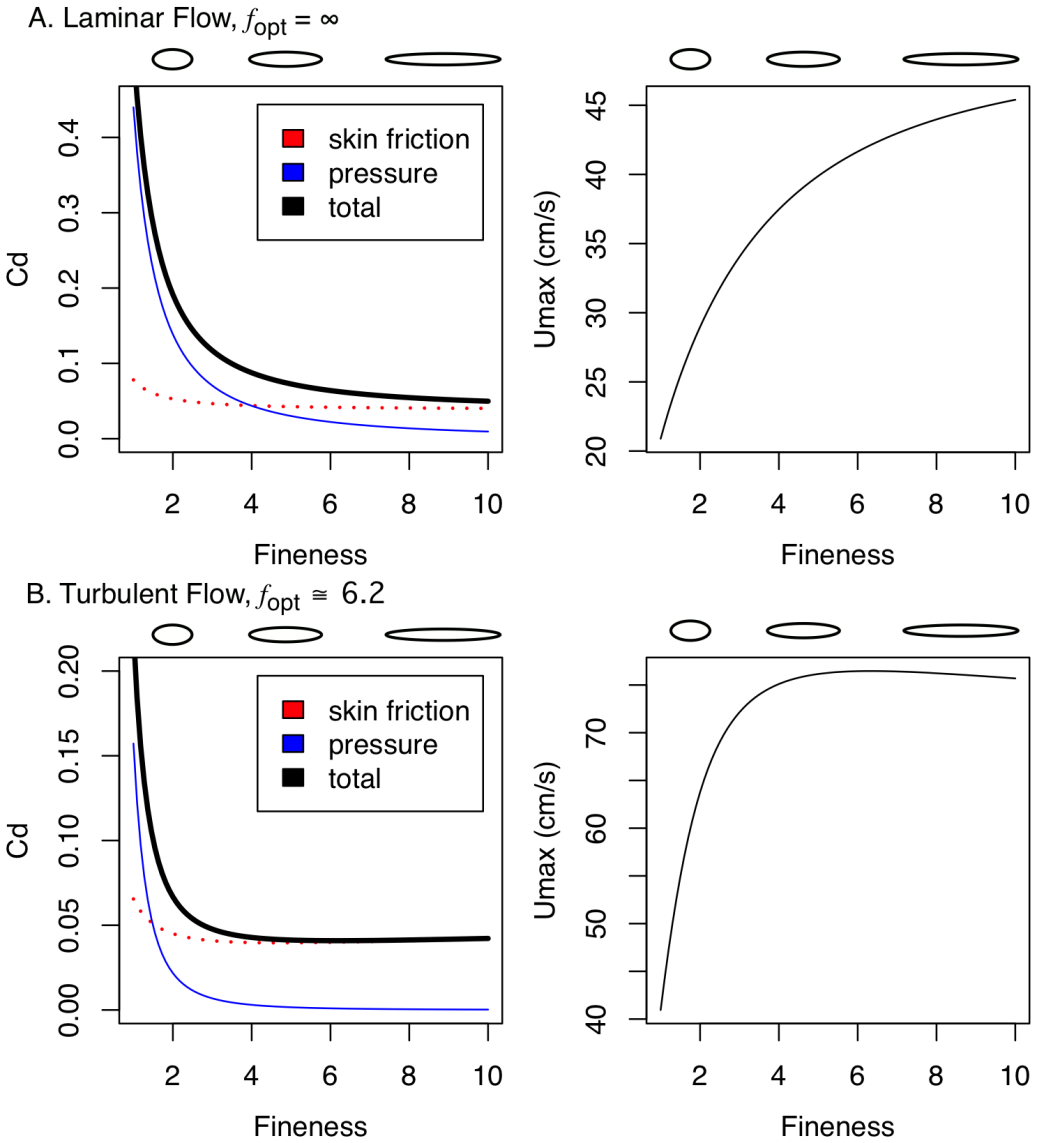
Performance as a function of fineness for rigid bodies of revolution. *C*_D_(*f*) (left panel) and modeled maximum-prolonged swimming speed (right panel) for laminar (A) and lower-end of turbulent (transitional) (B) flow. Drag coefficients are standardized using (*Vol*)^2/3^ as the reference area and computed for bodies of equal volume and speed, but differing Reynold's number (*Re*). Total (black line), skin friction (dotted red line) and pressure (thin blue line) components are illustrated. The elliptical figures above the plot are representative midline sections for finenesses of 2, 5, and 10 to show the relative length and depth of bodies of differing fineness but equal volume. The scale of the ordinate differs between (A) and (B) to emphasize the shape of the curve within each plot.

The shape of the function *C*_D_ (*f*), including the rate of increase of drag on either side of the optimal fineness, *f*_opt_, the location of *f*_opt_, and even the presence of an optimal fineness, is sensitive to several parameters, including scale of the fluid flow (Reynolds Number), how size is standardized (for example, by length, wetted area, or volume), and if *Re* or speed are held constant for size-standardized bodies (see Fig. 2). Consequently, there is not a universal, optimal fineness. Indeed, we use a function in which no *f*_opt_ exists (that is the function is monotonically decreasing with increasing *f*). The effect of fineness on drag as shape moves away from *f*_opt_ is especially noteworthy; for example, it has been noted [37], [38] that while *f*_opt_ occurs at 4.5 (for transitional flow and constant *Re*), only small differences (less than 10%) in drag occur between 3 and 7. For fishes in this fineness range, fineness-habitat associations may be dominated by other functional demands on body shape and fineness-performance associations may be difficult to detect when the expected signal (the slope of *C_D_*(*f*)) is small.

We use equations from Hoerner's classic compendium of empirical drag measures [33] to derive the relationship between the volume-specific drag coefficient, *C_D_*, and *f*. These equations, used in many previous models of the effect of shape on swimming performance [24], [38], [39], are based on estimates of drag measured on axially symmetric bodies. The fishes in this study all have transverse-section that are deeper than wide and, consequently, our model necessarily assumes the function *C_D_*(*f*) is invariant to eccentricity of the transverse section.

Predictions of the drag model depend critically on which equations are used but usage has been variable and confusing. For example, in his seminal work on fish swimming, which included a section focusing on the effects of *f* on swimming performance, Bainbridge [39] discussed the importance of standardizing by volume but actually used the equation for frontal-area standardization in the transitional regime (Hoerner eq. 6–31), which gives *f*_opt_ near 2.6. In his discussion of the role of *f* on swimming performance, Blake noted that *f*_opt_ is 4.5, which is true for the transitional regime, volume-specific equation (Hoerner eq. 6–36), but the only total-drag equation given was that for wetted-area standardization in the transitional regime (Hoerner eq. 6–28), which is asymptotic and does not contain an *f*_opt_.

For the fishes in this study, *Re* ranged from 6×10^3^ to 8×10^4^, which suggests a laminar boundary layer [36]. For data in the laminar flow regime, the volume-specific drag coefficient is derived using equations 6–24 and 6–35 from Hoerner [33]

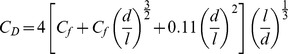
(3)where *C_f_* = 1.328*Re* ^−0.5^, *l* is body length, *d* is maximum diameter, and *Re* is computed using *l* as the reference length. For comparison, we also discuss the volume-specific drag coefficient for the transitional flow regime, which is modeled by eq. 6–36 in (Hoerner, 1965),
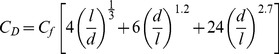
(4)where *C_f_* = 0.427[log10(*Re*)−0.407]^−2.64^ [33]. We note that using the derivative of eq. 4 to find the *f* that minimizes *C*_D_ yields *f*_opt_ = 4.6 (this minimum is effectively the standard *f*_opt_ used in the fish literature). However, if we are pursuing the question “which body fineness minimizes drag at speed *U*” (that is, we are developing a model of minimizing drag holding volume and speed constant), then *Re* must vary and we cannot simply use the derivatives of eqs. 3 and 4 to find *f*_opt_. To find the *f* that minimizes drag for a constant volume and swimming speed but that (necessarily) differ in *Re*, we computed *C_D_* using eqs. 3 and 4 for all bodies of equal volume and *f* ranging between 1 and 20 using 0.1 increments of *f*. Volume and velocity were set such that, for bodies with *f*≤10, the *Re* was between 10^4^ and 10^5^ for the laminar flow model and between 10^5^ and 10^6^ in the turbulent (or transitional) model. For the laminar flow model, the input volume and velocity were 0.000015 m^3^ and 0.6 m•s^−1^. For the transitional flow model, the input volume and velocity were 0.001 m^3^ and 1.5 m•s^−1^. Diameter was computed as 4*V*(0.65π*f*)^−1/3^ [33], where *V* is volume and 0.65 is the value of the prismatic coefficient, which is a measure reflecting the bluntness of the nose and tail of the modeled body. Length was computed as the product of fineness and diameter. The *C_D_* resulting from these modeled bodies of revolution that differ in fineness are shown in Fig. 2.

In contrast to the traditional drag model, bodies of constant volume and speed moving in the laminar flow regime display an asymptotic *C_D_*(*f)* with no *f*_opt_ (Fig. 2A). The asymptotic relationship indicates that the cost of *f* becomes very small as *f* increases. Consistent with the tradition drag model, *f*_opt_ exists in bodies of constant volume and speed moving in the transitional flow regime but the function is very flat above *f* = 3 (Fig. 2B). For our parameterization of volume and velocity, *f*_opt_ is 6.2. The *C_D_*(*f*) curves makes different predictions for fishes swimming in the laminar versus transitional flow regimes. For laminar flow, the drag model predicts monotonically increasing endurance swimming performance (*U*_max_) with *f*, with the slope becoming flatter at higher *f*. For transitional flow, the drag model predicts a performance peak at *f*_opt_ = 6.2 and, importantly a very small effect size (slope) at *f*≥4.

We parameterized eq. 2 using the simulated body shape data above and the body shape data of our fish. For mechanical efficiency, we use *η* = 0.34, the average of the peak efficiency for simulated rowing and flapping pectoral fins (0.09 and 0.59, respectively) [40]. We computed maximum power using 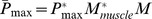, where the * indicates body mass specific (that is, the raw measure divided by body mass, *M*). For the simulated data, we used *M* = *Vρ*, where *ρ* is the density of water. For 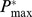, we used 16.5 WKg^−1^, the value reported for the pectoral fin muscles of the bluegill (*Lepomis macrochirus*) [41]. We used 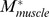 = 0.019 for relative muscle mass based on the mean muscle masses reported for the closely related pectoral fin swimmers in Thorsen and Westneat [42]. Following Jones et al. [41], we excluded the contributions of *m. arrector ventralis* and *m. adductor superficialis* to the mass of muscle that contributes to propulsive power. Mass-specific muscle power varies with fiber-type composition [43]. Consequently our value of mass-specific power may be high given that the bluegill muscle is composed of about 55% fast-glycolytic fibers [44] while the MPF swimmers in this study are likely to be dominated by slow-oxidative fiber types [45]. We are not too concerned about the precision of our parameterization as these values will predominantly affect the elevation of the function of *U*_max_(*f)*, which is not our goal, but will have very little effect on the shape of *U*_max_ (*f*), which is our goal.

The computation of *U*_max_ using eq. 2 required iteration since a velocity is required to compute *C*_D_ (specifically, it is need to compute *Re* in order to compute *C*_f_ in eqs. 3 and 4). We seeded the iteration with *U*_max_ = two body-lengths/s and used the output *U*_max_ as the input to the next iteration. Using a tolerance (the difference between input and output *U*_max_) of 0.001, the computation generally took about four iterations.

Modeled *U*_max_ as a function of fineness for MPF swimmers at 10^4^<Re<10^5^ (laminar) and 10^5^<Re<10^6^ (transitional to turbulent) are given in Fig. 2C and Fig. 2D. The slopes of *U*_max_(*f*) at different levels of fineness for both laminar and transitional flow regimes are tabled in Table 1. Qualitative predictions using *U*_max_ or *C*_D_ are the same but, because *U*_max_ is proportional to (*C*_D_)^−1/3^, the cost of drag is not as severe as when naively comparing *C*_D_. For the laminar model, *U*_max_(*f*) is monotonically increasing with no optimal fineness. For our parameterization of the transitional model, the optimal fineness is 6.3, which is slightly higher than the optimum that minimizes *C*_D_. Minimal exploration of eq. 2 suggests *f*_opt_ in the *Re* range 10^5–6^ will be ±0.1 unit from 6.3.

**Table 1:** Relative cost of change in fineness.

	Laminar		Transitional	
*f*	*α*	*α'*	*α*	*α'*
1	10.5	1.60	33.8	5.15
2	6.24	0.95	13.5	2.06
3	4.11	0.63	4.74	0.72
4	2.85	0.43	1.71	0.26
5	2.04	0.31	0.57	0.09
6	1.51	0.23	0.09	0.01
7	1.15	0.17	−0.12	−0.02
8	0.89	0.14	−0.23	−0.03
9	0.70	0.11	−0.27	−0.04
10	0.56	0.09	−0.29	−0.04

**Figure 3:**
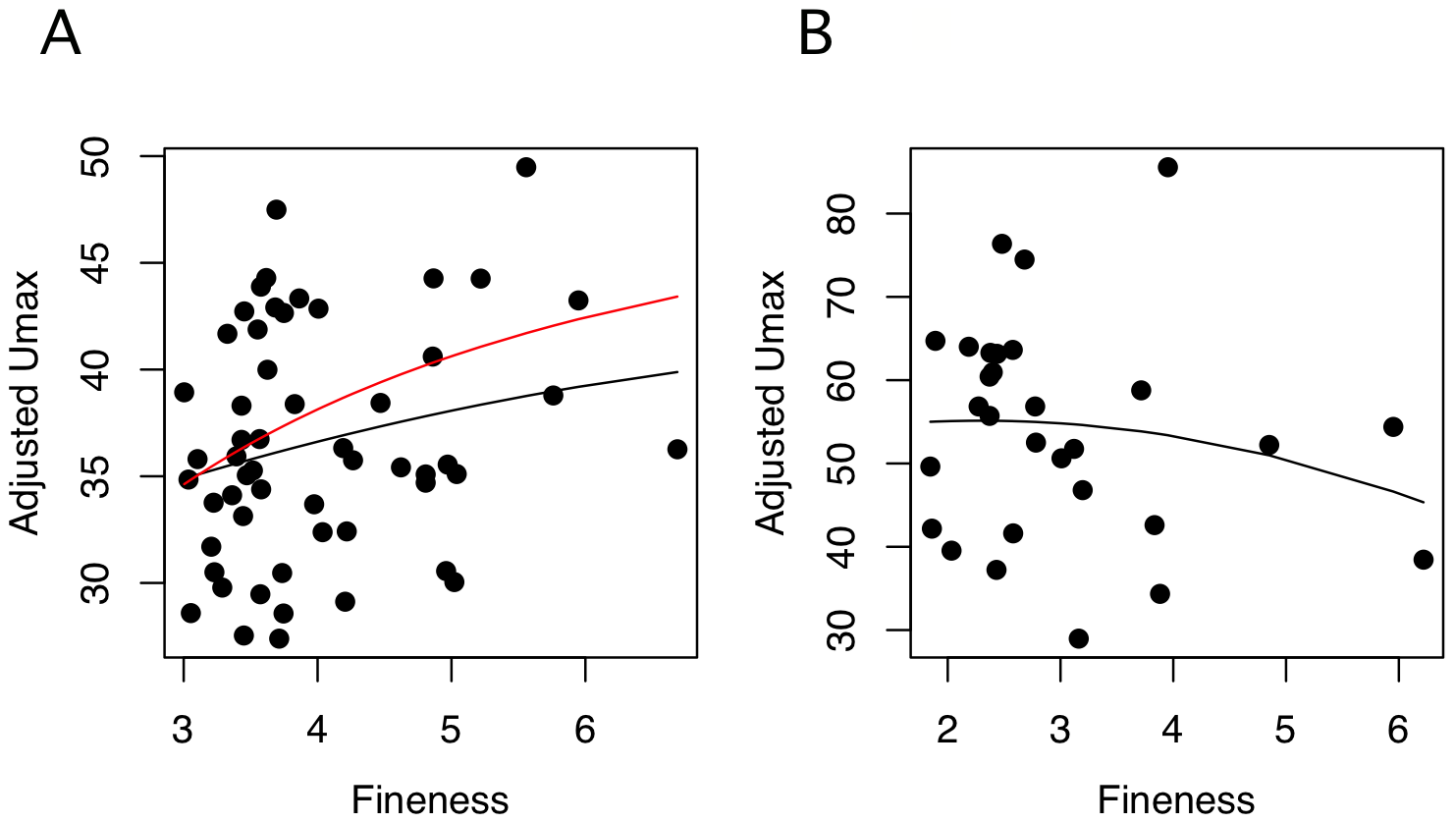
Maximum prolonged-swimming speed as a function of fineness in coral reef fishes. (A) pectoral-fin (MPF) and (B) body and caudal fin (BCF) subsets. The values are adjusted using the residuals from the regression of *U_max_* on *M* ^1/3^ and *AR* (fin aspect ratio). The red line is the modeled *U*_max_ (eq. 2). The black lines are the quadratic fit of the adjusted *U_max_* on fineness (*f*).

Raw (*α*) and standardized (*α'*) effects of fineness (*f*) on *U*_max_ for the laminar and transitional model of rigid-body drag. The coefficients are from a mechanistic and not regression model, and thus are truly causal (in the world of the model). The *ρ* aw coeffcients are the slopes of the curves in Fig. 3C, D at fineness 1–10. Except at *f* = 1, these approximately equal the percent increase in swimming speed given a unit increase in *f*. The standardized coefficients are standardized effect sizes represent the average change (or effect) in standard deviation units.

Again, the drag model assumes a rigid body propelled by an external motor. Throughout the range of prolonged swimming speeds, pectoral fin swimmers maintain very straight, rigid bodies that do not show any conspicuous passive undulation (known as flutter), at least in a laboratory water tunnel with laminar flow and while moving about the reef [2], [3], [5], [35], [46]. To a first approximation then, the boundary layer in MPF swimming fish should be similar to that on a rigid body-of-revolution and the drag model should be useful for understanding performance variation in MPF swimming fish. Computational fluid dynamic models of near body flow support this assumption [47].

### The drag-thrust model for body-caudal swimmers

For body-caudal fin (BCF) swimmers with self-propelled, undulatory bodies, in which the body acts as the source of thrust, normal forces, and parasite drag, an alternative model of optimal fineness that takes into account these additional forces should be more predictive the drag model. Unfortunately, no such model exists fully. Instead, we generate predictions using the results of two computational models of the effect of body shape on endurance swimming performance [23], [24]. Because the two published results do not comprehensively explore the shape of the fineness-performance function, our prediction for the BCF dataset is very general. Before summarizing the model and the prediction, we first show why the drag-model should not be very useful for BCF swimmers.

Optimal shape in the drag model is determined by the effect of fineness on skin-friction and form drag. For externally propelled, rigid bodies, optimal shapes are more elongate than spherical because the elongate, tapering body reduces form drag by moving the point of flow separation posteriorly. However, fishes that swim by BCF propulsion using axial undulation are self-propelled, undulating bodies. Flow over self-propelled, undulating bodies stays attached along the entire length of the body so the only component of drag is skin-friction [36], [48]–[52]. Indeed, in a self-propelled undulating body, the net pressure force over a stroke cycle is in the thrust (and not drag) direction [34], [50], [53], exactly opposite that on a rigid body propelled by external motors (either a towed body or a fish swimming by pectoral fins). In summary, there is no form drag in a self-propelled undulating body, at least over the body as a whole and over a complete stroke cycle. Instead, pressure “drag” contributes to thrust.

A mechanistic model of optimal fineness for BCF swimmers, then, must account for skin friction drag and pressure forces in the direction of swimming (thrust) and normal to the swimming axis, which contributes to wasted energy, reducing mechanical efficiency. Modeling this optimum is not trivial for multiple reasons. First, skin-friction in undulating bodies is elevated above that of rigid-bodies [36], [50], [54] and we have no simple model of the magnitude of this amplification as a function of the parameters controlling undulatory kinematics. Second, there is a substantial interaction between kinematic parameters and body shape on swimming performance [23], [31], [32]. The effect of this interaction on optimal modeling is exacerbated by the fact that undulatory kinematics will be a function of both internal stresses from muscle contraction and the deforming skeleton, including the skin, and external fluid stresses [55] and fineness will affect both internal and external stresses. Third, the space of optimal solutions needs to be limited by available muscle power [24]. Fourth, different aspects of endurance-swimming performance, such as efficiency (including Cost of Transport) and maximum sustained-swimming speed, have different optimal body shapes [23], [24], [31].

Our predictions for the causal relationship between fineness and endurance-swimming performance are generated from two computational models of the effects of body shape on endurance-swimming performance. Chung [23] used computational fluid dynamic (CFD) simulations to show that momentum capacity (essentially normalized swimming-speed) increases with fineness in fishes swimming with a continuous BCF gait, at least up to the maximum fineness (8.33) occurring in the fishes in the study. Chung's model did not account for either available muscle power or the effect of fineness on flexural stiffness of the body (and thus swimming kinematics) and, consequently, we do not know how the results might change given these inputs. In a large simulation optimizing body shape on endurance-swimming performance across a broad size range, Tokic and Yue [24] found that the optimal fineness for maximizing sustained swimming speed in fish in the size range of those in this study was higher than that occurring in our data. Importantly, Tokic and Yue's simulation modeled both internal and external stresses and limited solutions by available muscle power. Combined, both computational results suggest that we should find a positive effect of fineness on maximum endurance swimming speed but neither simulation provides the detail necessary to predict the shape of the function *U*_max_(*f*). That is, we do not have any prediction for how the effect of *f* on *U*_max_ should vary among levels of *f*. We note that these predictions differ from application of the drag model using transitional flow to BCF swimmers [56], which predicts an optimal fineness of 4.6 for bodies moving at equivalent *Re* or 6.2 for bodies of equivalent volume or mass.

### Swimming speed and morphometrics

Maximum prolonged-swimming speeds are from the published study by Fulton [3], where the methods of collection are described fully. Body morphometric data were collected from these same individuals but not published. Briefly, fishes from 84 species (55 MPF, 29 BCF) were collected on SCUBA on reefs near the Lizard Island Research Station using an ultra-fine monofilament barrier net, then transported to aquaria within 2 hrs of capture. After a minimum 3 hr still-water stabilization prior to testing, all individuals were speed tested within 36 hrs of capture. A stepwise, increasing-velocity test was used to estimate maximum prolonged swimming speed, *U*_max_, in both MPF and BCF swimmers. For the MPF swimmers, the transition speed, *U*_pc_, from steady MPF propulsion to an unsteady burst-and-glide BCF propulsion was used as the measure of *U*_max_. For the BCF swimmers, the critical swimming speed, *U*_crit,_ which is the maximum speed that could be maintained in the water tunnel, was used as the estimate of *U*_max_. Following the endurance-swimming speed test, the fish was anesthetized in 5% clove oil solution then ice-water slurry, weighed to the nearest 0.1 g, and body dimensions measured to the nearest 0.1 mm using dial calipers.

The fish in this (and most any) study have non-circular transverse sections and, consequently, fineness, *f*, differs in sagittal (lateral view) and coronal (dorsal view) planes. The frequent practice of measuring *f* in lateral view results in an increasingly misleading value of body shape relevant to drag and thrust production as the transverse section becomes more eccentric. For a more relevant value, we computed *f* as *l/d*_e_, where *l* is the standard length of the fish and *d*_e_ is the equivalent diameter of a circle of equal area (for MPF swimmers) [35] or equal perimeter (for BCF swimmers) [54] as the ellipse with major and minor axes equal to the maximum depth and breadth of the body. For the MPF swimmers, *d*_e_ is the geometric mean of depth and breadth, *d*_e_ = (*d*_max__*_ *b*_max_)^½^, which standardizes *d*_e_ by cross-sectional area and has the effect of giving more weight to the smaller input diameter. The geometric mean *d*_e_ assumes that the smaller diameter has more influence on flow separation behavior, such that very narrow fish will have less separation than expected if *f* were calculated with the arithmetic mean. For BCF swimmers, *d*_e_ is the elliptical mean, 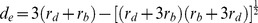, where *r_d_* and *r_b_* are 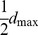 and 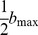. The elliptical mean standardizes by surface area and assumes that flow separation is effectively zero.

To standardize by a volumetric measure, we used total fish mass, *M*. The large effect of propulsive fin shape on prolonged swimming speeds [2], [5], [16] will confound results if fin shape is both statistically correlated with *f* and contributes to performance. Therefore, to adjust for fin shape, we used the aspect ratio (*AR*) of the pectoral fin (MPF subset) and caudal fin (BCF subset) as additional predictor variables. Fin AR was calculated from digitized images of amputated fins as 2*length^2^/area for pectoral fins and height^2^/area for caudal fins, where length and height were the leading edge length of a single pectoral fin or the vertical height from tip to tip of the caudal fin [2], [57], for a minimum of three replicate individuals per species (the pectoral fin AR is doubled to conform to the definition of AR for wings in the aerodynamics literature). All morphometric measures are given in Table S1.

### Statistical analysis

An “observational” model of effect of fineness on volume-specific endurance speed was investigated using multiple regression and model selection methods with log*U_max_* as the response variable and *f*, *f* ^2^, *AR*, and log*M* as the predictor variables. BCF and MPF datasets were analyzed separately. The variables were mean-centered and standardized to unit variance before entering into the multiple regression. The quadratic factor, *f* ^2^, was computed after centering *f* but before standardizing to ensure interpretability of the linear coefficient. We used a model selection approach to evaluate all combinations of the predictor variables (without interactions) and ordered model goodness-of-fit by the small-sample Akaike Information Criterion (AICc) (Hurvich & Tsai 1989). We retained all models with ΔAICc≤2 [58], [59], where ΔAICc is the difference between the model's AICc and the minimum AICc. Model-averaged beta coefficients were computed from the retained models using AICc as weights [58]. We estimated bootstrapped confidence intervals of the beta coefficients using simple percentiles of model-averaged coefficients from 4999 re-sampled pseudo-datasets. We also report whole-model adjusted *R* ^2^ as an easily interpretable measure of goodness-of-fit and supplement the bootstrap computed confidence intervals of each effect with the effect *P* value to guide our confidence in the effect estimate (we do not strictly interpret an arbitrary alpha as “significant” or “non-significant”). We used the statistical computing software R [60] for all statistics.

We used a combination of methods to adjust the degrees of freedom of the statistical tests due to phylogenetic autocorrelation of the residual error. We first estimated correlations among the predictor variables and between the predictor variables and *U*_max_ using phylogenetically independent contrasts [61] using the R package *ape* [62]. We used the *pgls* function from the R package *caper* [63] to estimate the beta coefficients using a phylogenetic generalized least squares (pGLS) regression [64], [65]. For each input model (different combinations of the predictors), we estimated the phylogenetic weighting parameter, λ, of the residuals using maximum likelihood. Lambda weights the effect of the expected covariance matrix (given the phylogeny) on the regression estimates [64], [65]. If the λ of the residuals is zero, the pGLS reduces to the ordinary least squares (OLS) regression. Revell [65] showed that the OLS is a better estimator if λ of the residuals is zero even if the λ of the input variables is large. Our phylogeny is taken from a time-calibrated megaphylogeny analysis of GenBank data for ray finned fishes (Rabosky et al, in review), pruned to match the species for which performance and morphometric data is available. Six species in the performance data set (Apogon nigrofasciatus, Heniochus singularis, Amblygobius decussatus, Scolopsis bilineatus, Cirrhilabrus punctatus, Pseudocheilinus hexataenia) were not present in the megaphylogeny. In each case we used another species from the same genus to represent this tip in our comparative analyses.

## Results

For our mechanistic model of *U*_max_ as a function of fineness, we parameterized eq. 2 using mean body volume (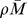) and the range of fineness from our MPF data and the values of *η* and 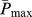 as for the simulated data. This modeled *U*_max_ is shown as the red line in Fig. 3A. The points in Fig. 3A are the measured *U*_max_ adjusted for *AR* and *M* ^1/3^ using OLS regression. The black line is the quadratic regression through these adjusted *U*_max_ (the “observational” model). The elevation of the modeled (red) curve is somewhat lucky as this elevation is very sensitive to the choice of *η* and 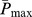. Again, it is the shape of the curve that we are concerned with. The slope (*α*) of *U*_max_(*f*) for *f* between 1 and 10 are tabled in Table 1. In addition to the raw slopes, standardized slopes are given, which were computed as 
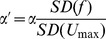
 where SD() indicates the standard deviation of *f* and adjusted *U*_max_ for our data.

A quadratic regression of the modeled *U*_max_ as a function of the mean-centered *f* and *f* ^2^ yields linear and quadratic coefficients of 2.95 and −0.39. A quadratic regression of the adjusted *U*_max_ yields coefficients of 1.59 (1SE: 1.23) and −0.15 (1SE: 0.89). The mechanistic model is covered by the 95% confidence intervals of the observational coefficients but so is a null model of no effect. To measure the goodness of fit of the shape, we re-centered the modeled *U*_max_ to have a mean equal to the mean adjusted *U*_max_. The percent of the total variance in adjusted *U*_max_ explained by the model is 0.015, which we computed as 
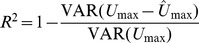
, where and is 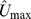 is the modeled *U*_max_. To obtain a probability of finding an *R* ^2^ of 0.015 under a null model of no relationship between *f* and *U*_max_, we permuted *U*_max_ 4999 times, recomputed *R* ^2^ for each permutation, and used the fraction, *g*, of 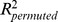
≥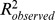 to compute the probability as 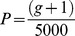. We found *P* = 0.054.

Within the MPF (pectoral fin propulsor) subset of species, we found generally moderate correlations among the predictor variables and between the predictor variables and log*U*_max_ for both the raw variables and the phylogenetically independent contrasts (PICs), with the exception of the correlation between pectoral fin aspect ratio (*AR*) and log*U*_max_, which was very high (Table 2). Notably, correlations among traits are similar using either the raw variables or the PICs, with the exception of log*M* and pectoral fin *AR*, which is moderately positive for the raw data but only trivially positive for the PIC data. Fineness (*f*) has a moderately positive correlation with pectoral fin *AR* and a moderately negative correlation with log*M*, while all three predictor variables have positive correlations with log*U*_max_.

**Table 2:** Correlations among predictor variables and between predictor variables and maximum prolonged swimming speed, *U*_max_.

a) MPF swimmers				
	*f*	*AR*	log*M*	log*U*_max_
*f*		0.25	−0.34	0.28
*AR*	0.33		0.01	0.8
log*M*	−0.23	0.39		0.2
log*U*_max_	0.34	0.89	0.47	

b) BCF swimmers				
	*f*	*AR*	log*M*	log*U*_max_
*f*		−0.6	−0.36	−0.17
*AR*	−0.50		0.51	0.31
log*M*	−0.15	0.31		0.38
log*U*_max_	−0.43	0.50	0.17	

The coefficients are the bivariate Pearson product-moment correlation among raw variables (below diagonal) and phylogenetically independent contrasts (above diagonal). The predictor variables are *f* (body fineness ratio), *AR* (propulsive fin aspect-ratio), and log*M* (body mass).

**Figure 4:**
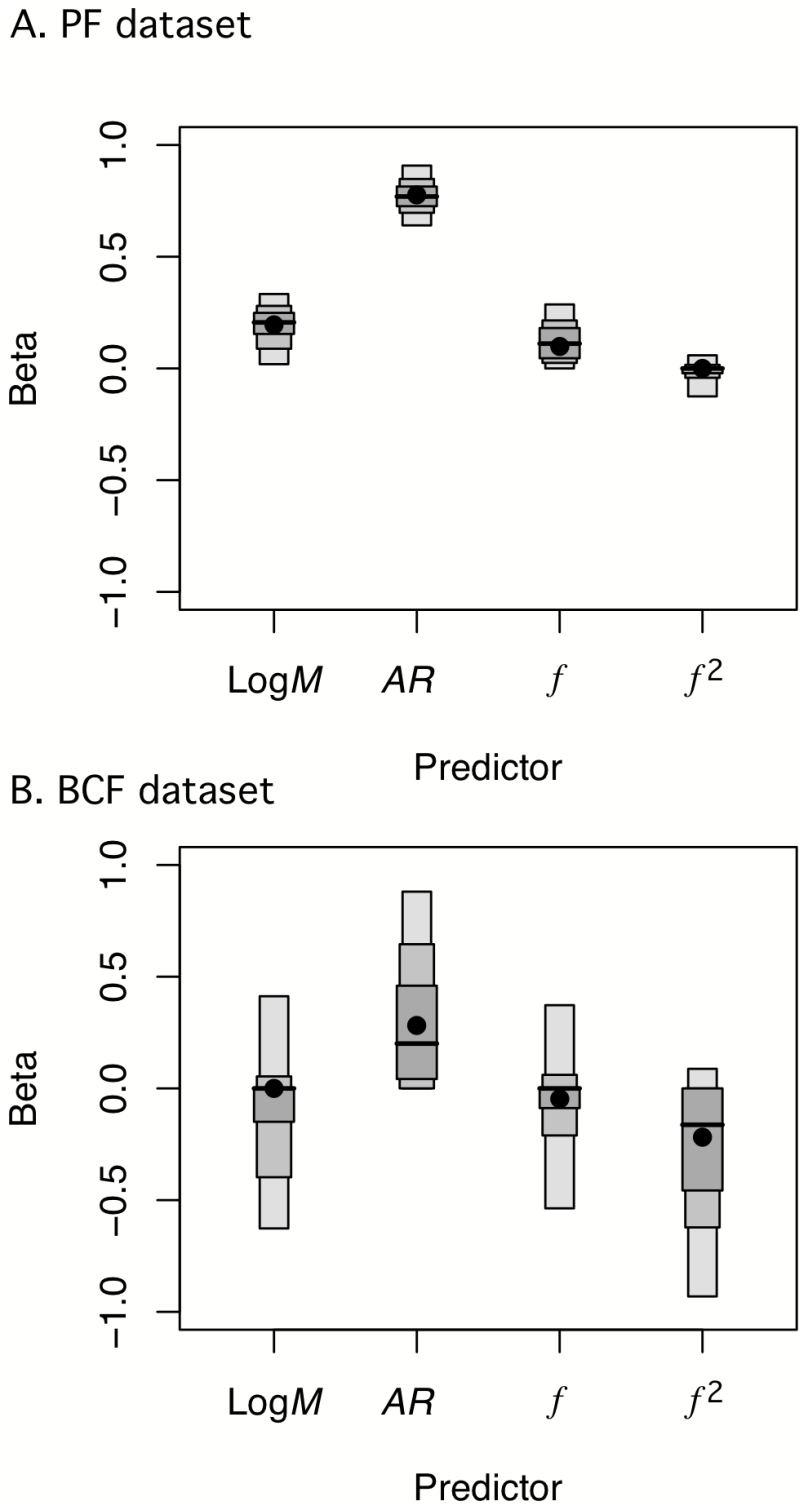
Box-percentile plots of the distribution of model-averaged *β* coefficients. The distribution is from 5000 bootstrap samples of maximum prolonged-swimming speed (log*U*_max_) regressed on the predictor variables. For each bootstrapped pseudosample, the model-averaged *β* coefficients were computed from all retained (AICc≥min(AICc)+2) models in which the variable was included in the model. The outer, intermediate, and inner boxes represent the 95%, 75%, and 50% confidence intervals, respectively. The dashed line represents the median and the dot represents the observed value (Table 3). The scale of the ordinate is the same between (A) and (B) to emphasize the greater variance in the estimates in the BCF subset. Predictor variables are log*M* (body Mass), *AR* (propulsive fin aspect-ratio), *f* (body fineness ratio), and *f* ^2^.

**Figure 5:**
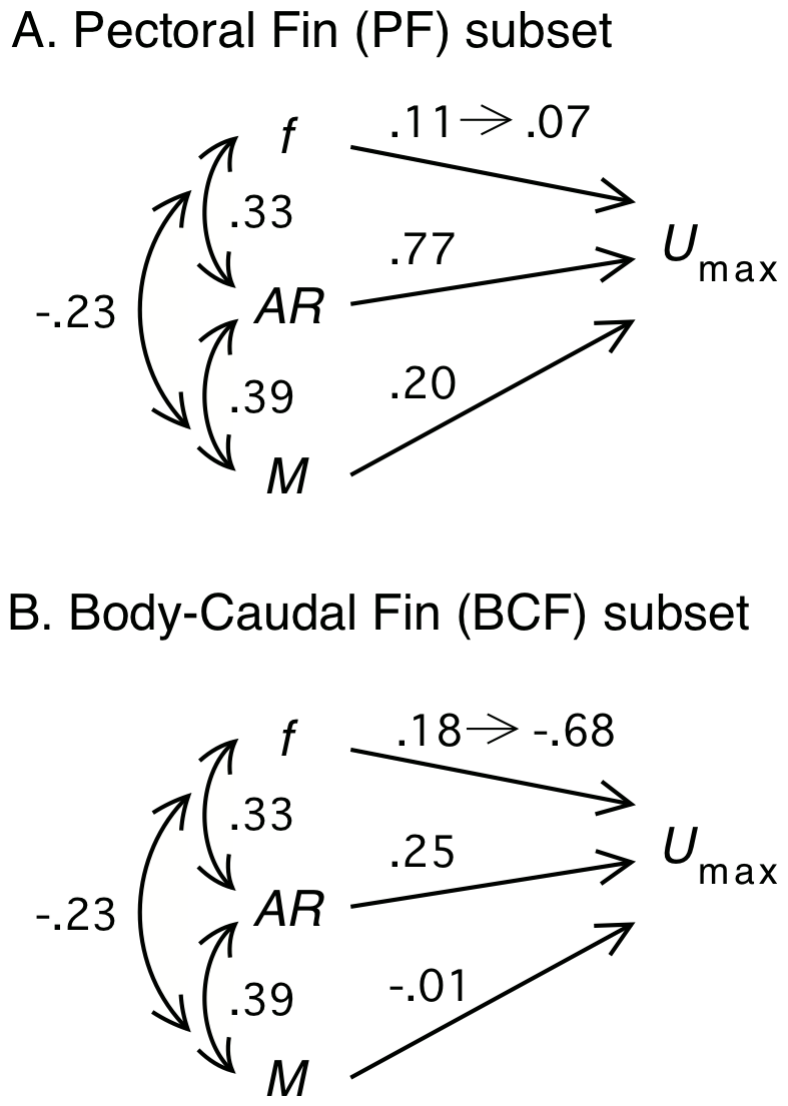
Path model of effect of predictor variables on maximum prolonged-swimming speed (log*U*_max_). (A) pectoral fin (MPF) and (B) body and caudal fin (BCF) subset of coral reef fish species. Predictor variables are *f* (body fineness ratio), *f* ^2^, *AR* (propulsive fin aspect-ratio), and log*M* (body mass). The linear and quadratic effects of *f* are combined to give the range of the effect from the *f*_min_ to *f*_max_. The *β* coefficient (the number above the directed path) for each predictor is the model-averaged (standard partial regression) coefficients as described in the text. Correlations among predictors are given next to the bi-directional path.

We used model selection and model averaging (as described in Methods) of the phylogenetic generalized least-squares (PGLS) regression of all combinations of predictor variables to create a phenomenological (statistical) model of the effects of aspect ratio (*AR*), mass (*M*), fineness (*f*), and *f* ^2^ on *U*_max_. For the MPF swimmers, two models were within 2 AIC units of the minimum AICc but we included the third best model, which was 2.14 units of the minimum AICc, in the computation of the model-averaged coefficients (Table 3). The best model included *f*, *AR*, and log*M* but not *f* ^2^. The second and third best model included *AR*, log*M*, and either both *f* and *f* ^2^ (model 3) or neither *f* and *f* ^2^ (model 2). Bootstrap confidence intervals for the coefficients are illustrated in Fig. 4. Model-averaged coefficients are illustrated with a path model in Fig. 5A. The model-averaged coefficient for *AR* (0.77) is very high for a standardized coefficient. log*M* has a moderate, positive effect (*β*_M_ = 0.2). The effect of *f* is small and positive and ranges from *β_f_* = 0.11 at *f*_min_ to *β_f_* = 0.07 at *f*_max_. All of the models including *AR* had effectively zero (*λ*<0.0001) phylogenetic signal in the model residuals. The *R* ^2^ of the model including all predictors was 0.819, which is very high for performance data and largely due to the effect of *AR* in the model. The *R* ^2^ of the model including only *AR* and log*M* was 0.804, which suggests that, as with the mechanistic model, fineness explains very little (∼0.015) of the variation in *U*_max_.

**Table 3:** Regression statistics of maximum prolonged-swimming speed on predictor variables.

MPF subset									
AICc	*β_f_*	*P*(*f*)	*β_f_*_2_	*P*(*f* ^2^)	*β_AR_*	*P*(*AR*)	*β*_log*M*_	*P*(log*M*)	AdjR^2^
70.07	0.14	0.051	-	-	0.76	∼0	0.21	0.004	0.81
71.88	-	-	-	-	0.82	∼0	0.15	0.025	0.80
72.21	0.17	0.067	−0.043	0.61	0.76	∼0	0.21	0.004	0.80

BCF subset									
AICc	*β_f_*	*P*(*f*)	*β_f_*_2_	*P*(*f* ^2^)	*β_AR_*	*P*(*AR*)	*β*_log*M*_	*P*(log*M*)	AdjR^2^
77.38	-	-	−0.50	0.006	-	-	-	-	0.22
77.47	-	-	-	-	0.50	0.006	-	-	0.22
77.79	-	-	−0.31	0.17	0.30	0.18	-	-	0.25
78.20	−0.24	0.21	-	-	0.38	0.06	-	-	0.24
79.39	-	-	−0.57	0.008	-	-	−0.13	0.51	0.21

Estimates from phylogenetic generalized least squares regression with *λ* estimated by maximum likelihood (*λ* is a parameter that controls the influence of the phylogenetic variance-covariance matrix on the estimates). In all models in both datasets, *λ* was less than 0.0001, indicating that the resulting GLS regression was reduced to an OLS regression. The *β* are standard partial regression coefficients, *P* is the probability of the effect. AICc is the small sample size corrected Akaike Information Criterion. AdjR^2^ is the R^2^ adjusted for the number of parameters in the model. The predictor variables are *f* (body fineness ratio), *f* ^2^, *AR* (propulsive fin aspect-ratio), and log*M* (body mass). The model-averaged regression coefficients are given in Fig. 5.

In the BCF (the body-and-caudal-fin propulsors) dataset, *f* has moderate to large, negative correlations with caudal fin *AR* and log*M* using either raw or PIC, while caudal fin *AR* and log*M* have moderate to moderately large positive correlation using raw data and PIC (Table 2). Body fineness *f* has a moderately large negative correlation with log*U*_max_, while caudal *AR* and log*M* have moderate to moderately large, positive correlations with log*U*_max_ using the raw data (Table 2). Using the PIC, the correlations between the predictors and log*U*_max_ have the same pattern as with the raw data but somewhat different magnitudes (Table 2).

For the BCF data, we have no mechanistic model to generate predicted swimming speeds to compare with the observations. Consequently, we compare the expected qualitative relationship of fineness and speed to the best statistical models. Four models have ΔAICc≤2 while a fifth (which we include in the model average) is within 2.01 of the minimum AICc (Table 3). Model-averaged coefficients are illustrated with a path model in Fig. 5B. The best three retained models are the three combinations of *f* ^2^ and *AR* (Table 3), with *f* ^2^ having a moderate to large negative quadratic effect and *AR* having a moderate to large positive effect on log*U*_max_. The linear effect of fineness is attenuated by the large quadratic effect (*β_f_*_2_ = −0.25); in the retained model with *f* but without *f* ^2^, the linear effect is −0.24. The effect of *f* ^2^ on log*U*_max_ is negative over the upper three-fourths of the range of *f* so that the linear effect of fineness goes from 0.18 at *f*_min_ to −0.68 at *f*_max_. The optimal fineness occurs at 2.77. All five best models had effectively zero phylogenetic signal (*λ*<0.0001) in the model residuals. The adjusted *R* ^2^ varies little (0.20–0.23) among the four best models. Unlike in the MPF subset of species, the estimates of the *β* coefficients for all predictors is highly variable across the bootstrap samples with all 95^th^ percentile boxes crossing zero except that for caudal fin *AR* in the BCF group (Fig. 4).

## Discussion

Our mechanistic model of *U*_max_ as a function of fineness for fishes powering maximum, prolonged-speeds using the median and/or pectoral fins (MPF swimmers) predicts different functions for fishes moving in the laminar versus transitional flow regimes. Given assumptions of the mechanistic model, we applied the model only to the subset of fishes that power swimming by oscillating pectoral fins (MPF swimmers) throughout their range of prolonged-swimming speeds. The laminar-regime mechanistic model predicted swimming speed about as well as an empirically fit regression. Indeed the shape of the mechanistic model and the statistical (observational) model (Fig. 3) are close in appearance. Nevertheless, both the mechanistic and statistical model explained only a small fraction of the variance in *U*_max_. For the fishes that power their highest prolonged-swimming speeds by axial undulation (BCF swimmers), we used multiple regression to generate a causal model and compared the direction of the coefficients to the results of published recent computational models of BCF swimming dynamics and energetics. These two modes of inferring causal association rely on very different sets of assumptions. In the following discussion, we address these points one by one.

### The drag model

Some version of the drag-model has been used repeatedly in the fish (and other animal) swimming literature, generally without much discussion on the scale of the focal fish relative to that of the drag model employed, or to the relevance of the shape of the function of drag (or *C*_D_) on fineness, or to the assumptions of the model relative to types of forces on swimming bodies. Exceptions include the observation that fineness should have only a small effect on drag (and swimming performance) over the middle to upper range of fineness in fishes [37], [38], the effect of high Reynolds Number (*Re*) on optimal fineness [66], and the explicit test of the theoretical optimal fineness using comparative performance data [56].

The shape of the function *U*_max_(*f*) is sensitive to the model of the drag coefficient (*C*_D_), which, in turn, depends on scale (or *Re*). We used a very simple model of scale effects in which a single function (eq. 3) was used for *Re* at the upper end of the laminar regime (10^4–5^) and a separate function (eq. 4) was used at the lower end of the turbulent (or transitional) regime (10^5–6^). The difference in how the effect (slope) changes with *f* between the two models is noteworthy. Few papers have explicitly cited a fineness optimum and those that have implicitly used a transitional-regime model to interpret swimming or habitat data despite the smaller scale of their focal fish [16], [56], [67], [68]. Our results show that these studies are generating a poor prediction by not modeling drag in the laminar regime. In the range of *Re* of the test fishes in these studies, no fineness optimum exists in the fineness range 1–10 (indeed, it does not exist at all). Our model of *U*_max_(*f*) in the transitional regime is qualitatively consistent with previous models [37], [38] of *C_D_*(*f*), that is, these functions are close to flat above *f* = 3 (Fig. 2, Table 1). By modeling *U*_max_ and not just *C_D_*, we can estimate the ability to detect an effect over some range of fineness. The standardized coefficients (Table 1) suggest that an effect of fineness should be readily detectable with moderate (N = 50–100) sample sizes for fishes swimming in the laminar regime, if the range includes fish with *f*<7. Even for fish in the fineness range 7–10, a fineness effect should be detectable with larger (N = 100–1000) sample size. By contrast, for fish swimming in the transitional regime, if the sample mostly contains individuals with *f*>4 (and especially *f*>5), the effect of *f* on *U*_max_ will be very hard to detect even with very large (N>1000) sample size. An assumption of any estimate of effect size from comparative data, of course, is that the estimate is not biased by unmeasured variables correlated with both fineness and swimming performance. We discuss this below in the conclusions.

### Fineness and prolonged swimming performance in pectoral fin swimmers

Within MPF swimmers, the relationship between *f* and endurance-swimming performance in the wild type threespine stickleback (*Gasterosteus aculeatus*) are largely consistent across studies and with the drag model: stickleback populations with finer bodies have higher endurance speeds [69]–[71]. However, these three studies are possibly confounded by the two-species comparison [72], and we note that inter-individual comparisons (which minimize problems of a two-species comparison) on lab-reared F1 stickleback have been ambiguous. For instance, Hendry et al. [73] found slight evidence for a *negative* relationship between fineness and prolonged-swimming performance among populations and within one of two populations. Conversely, Dalziel et al. [74] found evidence for a positive association between fineness and endurance performance within only one of two populations. When adjusting for multiple factors that might regulate endurance-swimming performance, however, Dalziel et al. [97] found very small effect sizes at the inter-individual level.

Our comparison of predicted *U*_max_ from the drag model with a large comparative dataset at a broad phylogenetic scale both minimizes issues of two-species comparisons and nicely complements the work on sticklebacks. Through comparing *U*_max_ over a wide range of *f*, we were able to more precisely test the drag model prediction of a curved performance surface. Despite the large standard errors of the estimated regression coefficients of *f* and, especially *f* ^2^, our statistically modeled curve is close to, but slighter flatter than, the mechanistically modeled curve (Fig. 3). Indeed, the large standard error of *β_f_*_2_ is very weak statistical evidence of a curved performance surface, despite the sign and magnitude of the coefficient close to its predicted value from the mechanistic model. In the sense that our statistical model and mechanistic model generated similar coefficients for *f* ^2^ despite the large standard errors, we were lucky. This effectively illustrates a difficulty with inferring causal relationships using comparative, observational data.

The ability to detect a quadratic effect of fineness depends on the range of fineness in the sample, since the signal (effect size) to noise (error from the statistical model) decreases with increasing fineness. The range of fineness, in turn, depends on our model mapping fineness in non-axisymmetric to axisymmetric bodies (that is, do we use the geometric, or elliptical, or some other mean of maximum breadth and depth as our diameter “equivalent” to a circle). To see this, we start with the assumption that the consequences of flow separation on a rigid body should become increasingly trivial as the transverse sectional depth to breadth ratio increases (a measure of frontal narrowness). Indeed, we would expect there to be a depth to breadth ratio above which the body acts more like a flat plate oriented parallel to the flow. We used the geometric mean of body depth and breadth to estimate the equivalent diameter of an axisymmetric body. The geometric mean weights the smaller dimension (breadth) more heavily, effectively shifting our fish to a “finer” shape than if the equivalent diameter were computed using the arithmetic mean. The magnitude of the shift increases with the eccentricity of the transverse section, such that very narrow fish bodies are expected to behave as very fine bodies regardless of the length to depth ratio. The right shift in fineness means that we would expect less of a quadratic effect than if we had used the arithmetic mean diameter. If the geometric mean undershifts the fineness (that is, the geometric mean does not weight the smaller radius enough), then even a perfectly estimated *f* and *f* ^2^ would appear underestimated relative to the mechanistic values. Given our standard errors, we cannot evaluate this component of error.

The preceding discussion assumes that the mechanistic model effectively captures the major features of the flow over a rigid fish body propelled by oscillating pectoral (or median) fins and how this flow changes with fineness. Differences between modeled and real flow could arise as a consequence of an interaction between the wake of the oscillating fins and the boundary layer on the body and how this interaction changes with fineness. Thrust-generating caudal fins of carangiform and thuniform swimmers create a jet that delays separation of the boundary layer or reattaches separated flow, severely depressing pressure drag [75], [76]. The consequences of this fin-induced delay in separation is the same as with a thrust-producing undulating body: except for extremely bluff bodies, pressure drag should be effectively eliminated. Unlike an oscillating caudal fin that is inline with the flow around the body, narrow-based pectoral fins generate most of the thrust distally well-outside of the body's boundary layer [47], [77]. As a first approximation then, the near-body flow over a fish propelling a rigid body with pectoral fins should be similar to that over a rigid body of revolution, which suggests that *f* should be able to predict relative swimming performance if unmeasured traits do not mask this relationship.

Given that finer bodies are more optimal for faster prolonged swimming speeds using MPF propulsion, the relatively few MPF fishes with a high *f* raises the possibility that functional trade-offs are limiting the repeated evolution of a high *f* phenotype. Fineness potentially affects many performance traits [4] that contribute to fitness on a coral reef, including the ability to burrow in reef sediment, maneuver among complex coral structure [78], signal to mates and competitors, and avoid predators while responding to rapidly changing hydrodynamic conditions [79]. In order to understand the complexity of how these functional demands shape the evolution of diversity of fish body shapes on a coral reef, we need better models of the functional consequences of body shape variation in combination with empirical data obtained under both laboratory and field conditions that acknowledge the biological and ecological contexts within which species are operating (including aspects such as foraging mode and reproductive status).

### Fineness and endurance-swimming performance in the BCF swimmers

The idea that a more streamlined or fusiform body reduces drag during steady, rectilinear swimming and consequently, increases endurance-swimming performance, permeates the literature on fish functional ecology and evolution [12]–[22], [68], [70], [80]–[85]. In support of this idea is the relatively consistent association between more optimal fineness and high-flow or open-water habitat [56], [86] and between endurance performance and open-water habitat [7]. In support of these patterns, recent computational modeling of self-propelled, undulating fish bodies predict a positive effect of fineness on maximum prolonged-swimming speed across the range of *f* in this study, although available simulation results do not allow us to predict the size or shape of this effect across the range of *f*. Nevertheless, our comparative results our inconsistent with these predictions and with the habitat-shape associations. Indeed, our results give moderate evidence for a negative association between fineness and maximum prolonged-swimming speed across most (if not all) of the range of fineness among the BCF swimmers in our data. Interestingly, our results are consistent with other direct comparisons of fineness and prolonged-swimming performance. Unfortunately, in the only other comparison at a broad phylogenetic scale, Fisher & Hogan [16] showed that fineness adds little to the ability to predict prolonged-swimming speed after adjusting other morphometric measures in juvenile reef fishes that comprise both MPF and BCF swimmers but do not give the value of the regression coefficient. Comparisons among individuals within a population or among ecotypes within a species have found, with few exceptions [87], either trivially small associations or a negative associations between fineness and endurance-swimming performance [13], [83], [84], [88]–[90]. These results suggest that any causal mechanism linking fineness and habitat in BCF swimming fishes may not be via the effect of fineness on prolonged-swimming performance.

### Conclusions

A major goal of much of comparative methodology is to infer function, or the effect of morphology on performance, using the sign and magnitude of regression coefficients. Unfortunately, regression is not up to this task except in extremely limiting cases. If a regression model fails to include all underlying variables that are both correlated with the measured variables and causally associated with performance via some path other than the measured variables, the regression coefficients of the measured variables are biased. This omitted-variable (or specification) bias is addressed extensively in the econometrics and epidemiology literature [91]–[93] but is, at best, perfunctorily acknowledged in the plant and animal function literature (or the ecology and evolution literature more generally) [94], [95]. The bias can both mask (drive coefficients toward zero) and augment (move coefficients away from zero) real effects [96]. Adding more variables to the model can increase as well as decrease the bias. Consequently, while some suggest that some information is better than none [94], in fact it's not if one's goal is causal interpretation of the coefficients. The value of the combination of comparative data and some mechanistic model, then, is not as an empirical “test” or “validation” of a model, which it cannot do, but as means of empirically quantifying how much variation in some trait (such a endurance-swimming performance) can be explained by one or more causal factors.

For our MPF dataset, fineness explains only a small fraction of the variation in size-specific endurance swimming performance in fishes swimming by pectoral fin propulsion even after adjusting for body size and pectoral fin aspect ratio. This pattern suggests, not surprisingly, that *U*_max_ is determined by multiple underlying factors, including unmeasured traits such as cardiac ventricle size, gill surface area, and various properties of the pectoral fin muscle including gearing, size, and enzyme activities [97]. If many factors affect function and these factors are not highly correlated with each other then the standardized effect size of most of these factors must be small (<0.1). While small effects are difficult to detect, they have evolutionary if not ecological relevance since very small selection differentials operating over thousands of generations can easily move mean phenotypes several standard deviations from some starting value [98]. For MPF fishes moving in the transitional regime, much of the performance space (*U*_max_(*f*)) is shallow enough that drag minimization during steady swimming should have very little influence on the direction of evolution of body shape.

## Supporting Information

Table S1 Morphometric and performance data for the 55 pectoral fin swimmers and 29 body-and-caudal fin swimmers.
(DOCX)
