# Supplementary material for: Body Fineness Ratio as a Predictor of Maximum Prolonged-Swimming Speed in Coral Reef Fishes
Source: PLoS One. 2013 Oct 18;8(10):e75422. doi: 10.1371/journal.pone.0075422 (PMC3799785; doi:10.1371/journal.pone.0075422)
Supplement: Table S1 — Morphometric and performance data for the 55 pectoral fin swimmers and 29 body-and-caudal fin swimmers. (DOCX) [file pone.0075422.s001.docx]

Table S1. Morphometric and performance data for the 55 pectoral fin swimmers and 29 body-and-caudal fin swimmers.

| Species | *Mass* (g) | *SL* (cm) | *D*_max_ (cm) | *B*_max_ (cm) | *U*_max_ (cm/s) | *f* | *AR* |
| --- | --- | --- | --- | --- | --- | --- | --- |
| (a) Pectoral Fin |  |  |  |  |  |  |  |
| *Acanthurus blochii* | 156.98 | 13.86 | 7.06 | 2.31 | 44.65 | 3.43 | 2.84 |
| *Acanthurus dussumieri* | 165.70 | 14.48 | 7.50 | 2.32 | 49.69 | 3.47 | 3.27 |
| *Acanthurus nigrofuscus* | 23.04 | 8.27 | 4.04 | 1.33 | 53.14 | 3.57 | 3.53 |
| *Acanthurus olivaceus* | 164.00 | 13.50 | 6.12 | 2.51 | 46.84 | 3.44 | 3.21 |
| *Acanthurus triostegus* | 130.68 | 14.08 | 7.14 | 2.51 | 61.86 | 3.33 | 3.64 |
| *Ctenochaetus binotatus* | 31.57 | 8.00 | 3.77 | 1.21 | 39.13 | 3.75 | 3.14 |
| *Ctenochaetus striatus* | 79.77 | 9.03 | 4.63 | 1.38 | 44.41 | 3.57 | 3.35 |
| *Anampses neoguinaicus* | 15.23 | 8.27 | 2.61 | 1.11 | 45.07 | 4.86 | 2.79 |
| *Bodianus mesothorax* | 47.07 | 11.73 | 3.98 | 1.73 | 32.10 | 4.47 | 2.04 |
| *Cheilinus chlorurus* | 41.90 | 9.97 | 3.82 | 1.62 | 34.69 | 4.01 | 1.93 |
| *Cheilinus fasciatus* | 51.56 | 12.77 | 5.47 | 2.33 | 36.33 | 3.58 | 1.96 |
| *Choerodon fasciatus* | 112.47 | 14.20 | 5.87 | 2.49 | 36.34 | 3.71 | 2.94 |
| *Cirrhilabrus punctatus* | 11.90 | 7.90 | 2.37 | 1.07 | 43.30 | 4.96 | 3.33 |
| *Coris schroederi* | 19.00 | 9.03 | 2.65 | 1.22 | 37.35 | 5.02 | 2.96 |
| *Epibulus insidiator* | 50.49 | 11.13 | 4.72 | 1.76 | 40.43 | 3.86 | 2.25 |
| *Gomphosus varius* | 21.53 | 12.07 | 2.82 | 1.46 | 57.09 | 5.95 | 3.37 |
| *Halichoeres margaritaceus* | 11.48 | 9.30 | 2.67 | 1.19 | 47.96 | 5.22 | 2.76 |
| *Halichoeres melanurus* | 6.14 | 7.13 | 1.99 | 0.77 | 33.46 | 5.76 | 2.21 |
| *Hemigymnus melapterus* | 29.25 | 9.93 | 3.83 | 1.63 | 36.17 | 3.97 | 2.63 |
| *Labrichthys unilineatus* | 13.03 | 8.00 | 2.43 | 1.14 | 24.26 | 4.81 | 1.83 |
| *Labroides dimidiatus* | 5.05 | 7.30 | 1.37 | 0.87 | 49.89 | 6.69 | 3.42 |
| *Macropharyngodon negrosensis* | 14.53 | 8.20 | 2.72 | 1.07 | 39.04 | 4.81 | 2.79 |
| *Oxycheilinus digrammus* | 43.83 | 12.00 | 3.71 | 1.57 | 27.54 | 4.97 | 1.94 |
| *Pseudocheilinus hexataenia* | 2.47 | 4.40 | 1.51 | 0.60 | 23.61 | 4.62 | 1.82 |
| *Stethojulis bandanensis* | 16.40 | 8.07 | 2.35 | 1.17 | 70.09 | 4.87 | 4.15 |

| Species | *Mass* (g) | *SL* (cm) | *D*_max_ (cm) | *B*_max_ (cm) | *U*_max_ (cm/s) | *f* | *AR* |
| --- | --- | --- | --- | --- | --- | --- | --- |
| *Thalassoma janseni* | 18.27 | 9.65 | 2.43 | 1.24 | 71.65 | 5.56 | 3.91 |
| *Thalassoma lunare* | 28.35 | 10.17 | 2.77 | 1.47 | 51.88 | 5.04 | 3.54 |
| *Abudefduf sexfasciatus* | 19.00 | 7.47 | 3.67 | 1.32 | 41.90 | 3.39 | 2.88 |
| *Abudefduf vaigiensis* | 9.40 | 5.90 | 2.87 | 0.98 | 45.00 | 3.52 | 3.15 |
| *Abudefduf whitleyi* | 41.20 | 8.92 | 4.68 | 1.63 | 44.49 | 3.23 | 3.34 |
| *Acanthochromis polyacanthus* | 25.81 | 8.80 | 4.48 | 1.60 | 39.40 | 3.29 | 3.09 |
| *Amblyglyphidodon curacao* | 31.36 | 7.98 | 4.90 | 1.44 | 36.27 | 3.00 | 2.30 |
| *Amphiprion akindynos* | 7.05 | 5.35 | 2.64 | 0.96 | 15.00 | 3.36 | 1.33 |
| *Chromis atripectoralis* | 8.50 | 5.98 | 2.68 | 0.91 | 43.42 | 3.83 | 2.86 |
| *Chromis weberi* | 18.65 | 7.83 | 3.26 | 1.38 | 47.69 | 3.69 | 2.51 |
| *Chrysiptera brownriggi* | 3.77 | 4.80 | 1.75 | 0.74 | 26.70 | 4.22 | 2.20 |
| *Chrysiptera rex* | 3.73 | 4.70 | 1.70 | 0.74 | 25.24 | 4.19 | 1.86 |
| *Chrysiptera rollandi* | 2.33 | 3.85 | 1.66 | 0.64 | 17.91 | 3.74 | 1.78 |
| *Chrysiptera talboti* | 1.70 | 3.53 | 1.47 | 0.52 | 18.49 | 4.04 | 1.70 |
| *Dascyllus aruanus* | 3.70 | 4.10 | 2.18 | 0.75 | 20.24 | 3.21 | 1.83 |
| *Dascyllus reticulatus* | 5.22 | 4.73 | 2.94 | 0.79 | 29.57 | 3.10 | 2.16 |
| *Dischistodus melanotus* | 35.88 | 9.43 | 4.12 | 1.71 | 28.69 | 3.55 | 1.62 |
| *Neoglyphidodon melas* | 19.80 | 7.50 | 3.62 | 1.32 | 30.24 | 3.43 | 1.98 |
| *Neoglyphidodon nigroris* | 20.73 | 7.50 | 3.84 | 1.41 | 26.67 | 3.22 | 2.04 |
| *Neopomacentrus azysron* | 3.87 | 4.90 | 1.74 | 0.78 | 27.16 | 4.21 | 2.44 |
| *Plectroglyphidodon lacrymatus* | 14.87 | 6.47 | 3.27 | 1.39 | 25.38 | 3.03 | 1.91 |
| *Pomacentrus amboinensis* | 10.42 | 6.43 | 2.87 | 1.21 | 15.00 | 3.45 | 1.73 |
| *Pomacentrus bankanensis* | 8.20 | 5.77 | 2.46 | 1.03 | 30.45 | 3.62 | 1.93 |
| *Pomacentrus brachialis* | 8.57 | 5.80 | 2.71 | 0.97 | 25.40 | 3.58 | 1.96 |
| *Pomacentrus chrysurus* | 11.17 | 6.37 | 2.77 | 1.12 | 35.22 | 3.62 | 1.95 |
| *Pomacentrus coelestis* | 2.94 | 4.40 | 1.50 | 0.71 | 30.81 | 4.26 | 2.26 |
| *Pomacentrus lepidogenys* | 7.68 | 5.57 | 2.40 | 0.92 | 35.94 | 3.75 | 2.11 |
| *Pomacentrus nagasakiensis* | 11.07 | 6.37 | 2.67 | 1.12 | 31.75 | 3.68 | 1.81 |
| Species | *Mass* (g) | *SL* (cm) | *D*_max_ (cm) | *B*_max_ (cm) | *U*_max_ (cm/s) | *f* | *AR* |
| *Premnas biaculeatus* | 16.23 | 7.03 | 3.37 | 1.23 | 16.70 | 3.45 | 0.85 |
| *Stegastes apicalis* | 44.93 | 9.38 | 4.72 | 2.00 | 21.81 | 3.05 | 2.02 |
|  |  |  |  |  |  |  |  |
| (b) Body and Caudal Fin |  |  |  |  |  |  |  |
| *Naso brevirostris* | 37.07 | 11.13 | 5.08 | 1.32 | 55.52 | 3.20 | 3.28 |
| *Zebrasoma scopas* | 49.89 | 10.14 | 6.05 | 1.62 | 62.66 | 2.44 | 2.55 |
| *Chaetodon aureofasciatus* | 21.63 | 6.80 | 5.45 | 1.14 | 47.35 | 1.86 | 2.97 |
| *Chaetodon auriga* | 24.30 | 8.47 | 5.30 | 1.17 | 56.49 | 2.37 | 2.62 |
| *Chaetodon baronessa* | 26.73 | 7.62 | 6.22 | 1.12 | 55.55 | 1.85 | 3.04 |
| *Chaetodon citrinellus* | 19.64 | 7.73 | 4.56 | 1.15 | 77.51 | 2.48 | 2.64 |
| *Chaetodon ephippium* | 21.45 | 7.95 | 4.96 | 1.13 | 64.26 | 2.37 | 2.86 |
| *Chaetodon kleinii* | 16.43 | 7.25 | 4.71 | 1.08 | 57.81 | 2.27 | 2.62 |
| *Chaetodon lunulatus* | 29.90 | 8.73 | 5.34 | 1.32 | 58.51 | 2.40 | 2.37 |
| *Chaetodon plebius* | 17.77 | 7.47 | 4.17 | 1.21 | 61.46 | 2.58 | 2.37 |
| *Chaetodon rainfordii* | 16.93 | 6.30 | 4.96 | 1.05 | 68.40 | 1.89 | 2.84 |
| *Chaetodon trifascialis* | 45.17 | 8.84 | 4.73 | 1.40 | 75.79 | 2.68 | 2.69 |
| *Chaetodon ulietensis* | 63.40 | 9.35 | 5.65 | 1.37 | 38.56 | 2.43 | 2.71 |
| *Chaetodon vagabundus* | 54.80 | 10.45 | 7.10 | 1.54 | 65.45 | 2.19 | 2.71 |
| *Chelmon rostratus* | 53.33 | 11.53 | 7.26 | 1.43 | 62.87 | 2.38 | 2.56 |
| *Heniochus singularis* | 14.10 | 6.60 | 4.87 | 0.93 | 32.54 | 2.03 | 1.97 |
| *Amblygobius decussatus* | 6.65 | 7.20 | 1.34 | 0.96 | 23.45 | 6.22 | 1.30 |
| *Valenciennea strigata* | 13.06 | 8.88 | 1.75 | 1.21 | 39.97 | 5.95 | 1.37 |
| *Lutjanus carponotatus* | 43.20 | 11.10 | 4.08 | 1.63 | 59.25 | 3.71 | 2.62 |
| *Scolopsis bilineatus* | 26.93 | 9.63 | 3.31 | 1.47 | 50.20 | 3.88 | 3.84 |
| *Centropyge bicolor* | 25.08 | 7.85 | 3.95 | 1.40 | 49.46 | 2.77 | 1.96 |
| *Centropyge vrolikii* | 30.70 | 8.30 | 4.48 | 1.61 | 40.06 | 2.58 | 2.44 |
| *Stegastes nigricans* | 43.13 | 9.43 | 4.59 | 1.91 | 53.48 | 2.78 | 2.66 |
| *Cephalopholis boenak* | 72.37 | 13.27 | 4.54 | 2.17 | 34.06 | 3.83 | 1.92 |
| *Plectropomus leopardus* | 60.77 | 13.73 | 3.61 | 1.92 | 49.14 | 4.85 | 2.35 |
| Species | *Mass* (g) | *SL* (cm) | *D*_max_ (cm) | *B*_max_ (cm) | *U*_max_ (cm/s) | *f* | *AR* |
| *Siganus corallinus* | 27.57 | 9.47 | 4.42 | 1.17 | 56.32 | 3.12 | 2.93 |
| *Siganus doliatus* | 26.23 | 9.53 | 4.66 | 1.12 | 56.44 | 3.01 | 3.03 |
| *Siganus spinus* | 62.20 | 13.40 | 4.66 | 1.80 | 85.67 | 3.95 | 2.61 |
| *Siganus vulpinus* | 96.63 | 13.47 | 6.06 | 1.91 | 29.37 | 3.16 | 2.66 |
